# A circulating exosomal microRNA panel as a novel biomarker for monitoring post‐transplant renal graft function

**DOI:** 10.1111/jcmm.15861

**Published:** 2020-09-11

**Authors:** Yimeng Chen, Xu Han, Yangyang Sun, Xiaozhou He, Dong Xue

**Affiliations:** ^1^ Department of Urology The Third Affiliated Hospital of Soochow University Changzhou China

**Keywords:** biomarker, estimated glomerular filtration rate, exosomes, kidney transplant, MicroRNAs

## Abstract

Accurate and effective biomarkers for continuous monitoring of graft function are needed after kidney transplantation. The aim of this study was to establish a circulating exosomal miRNA panel as non‐invasive biomarker for kidney transplant recipients. Plasma exosomes of 58 kidney transplant recipients and 27 healthy controls were extracted by gel exclusion chromatography and characterized by transmission electron microscopy, nanoparticle tracking analysis and Western blotting. Post‐transplant renal graft function was evaluated by estimated glomerular filtration rate (eGFR). Quantitative real‐time polymerase chain reaction was used to determine the expression of exosomal microRNAs (miRNAs). Exosomal miR‐21, miR‐210 and miR‐4639 showed negative correlations with eGFR in the training set and were selected for further analysis. In the validation set, miR‐21, miR‐210 and miR‐4639 showed the capability to discriminate between subjects with chronic allograft dysfunction (eGFR < 60 mL/min/1.73 m^2^) and those with normal graft function (eGFR > 90 mL/min/1.73 m^2^). Three‐miRNA panel exhibited higher accuracy compared with individual miRNAs or double indicators. One‐year follow‐up revealed a stable recovery of allograft function in subjects with low calculated score from three‐miRNA panel (below the optimal cut‐off value). In conclusion, a unique circulating exosomal miRNA panel was identified as an effective biomarker for monitoring post‐transplant renal graft function in this study.

## 1 INTRODUCTION

Since the first successful kidney transplant in 1954, kidney transplantation has become the routine management for patients presenting with end‐stage renal disease.^1^ The application of highly effective immunosuppressive drugs over the past 20 years has significantly improved the 1‐year survival of kidney grafts.^2^ However, chronic allograft dysfunction, which may due to both immunologic and non‐immunologic factors, is the major cause of renal allograft loss in the long‐term.^3^, ^4^ Thus, accurate assessment and monitoring of allograft function is critical for kidney transplant recipients.

Currently, measurements of serum creatinine (Cr), estimated glomerular filtration rate (eGFR) and proteinuria are often applied for the evaluation of progression of kidney injury.^5^ The gold standard test is histological diagnosis with a renal transplant biopsy.^6^ However, there are some disadvantages of low specificity and sensitivity or invasiveness during the evaluation. Cr is derived from the non‐enzymatic dehydration of skeletal muscle creatine, which itself is generated from amino acids in the liver.^7^ Thus, numerous factors such as muscle mass and turnover, sex, diet, race, liver function and medication use can influence serum Cr concentration.^8^ In kidney transplant recipients, serum Cr concentration can be affected due to the long‐term use of corticosteroids, infection, acute rejection and previous prolonged haemodialysis therapy.^7^ As a result, the Cr‐based eGFR estimation equation is also flawed, and the evaluation equation itself is not perfect.^9^ Moreover, proteinuria can be affected by exercise and diet.^10^ Kidney biopsies are considered to be the gold standard for evaluating allograft dysfunction. However, renal biopsy cannot be used to monitor the progression of injury because it is an invasive procedure and cannot be performed serially.^11^ Furthermore, the histological evaluation of biopsies is subjective and samples removed from one segment of the transplanted kidney may not represent the whole graft.^12^ Therefore, it is necessary to find a sensitive and non‐invasive biomarker for the continuous monitoring of graft function after kidney transplantation.

MicroRNAs (miRNAs) are a group of small non‐coding RNAs that can regulate up to 60% of gene expression in mammals by binding to the 3′ untranslated region (3′‐UTR) of the target messenger RNA (mRNA) involved in many diseases.^13^ Exosomes are small (40‐160 nm) membrane vesicles of endocytic origin that are released into the extracellular environment on fusion of multivesicular bodies with the plasma membrane.^14^, ^15^ Exosomal miRNAs are proved to be stably expressed in serum, plasma, urine, saliva and other body fluids.^16^ Many studies have indicated that levels of exosomal miRNAs are associated with renal function. For instance, the differential expression of five miRNAs (miR‐32, miR‐107, miR‐142‐3p, miR‐204 and miR‐211) in patients with chronic allograft dysfunction was confirmed by using an independent set of kidney tissue samples and paired urine samples.^17^ Moreover, five miRNAs (miR‐200b, miR‐375, miR‐423‐5p, miR‐193b and miR‐345) were identified as potential biomarkers for monitoring allograft function in the urine samples of renal transplant recipients.^18^ However, the role of circulating exosomal miRNAs in the monitoring of post‐transplant renal graft function has not been fully figured out.

**FIGURE 1:**
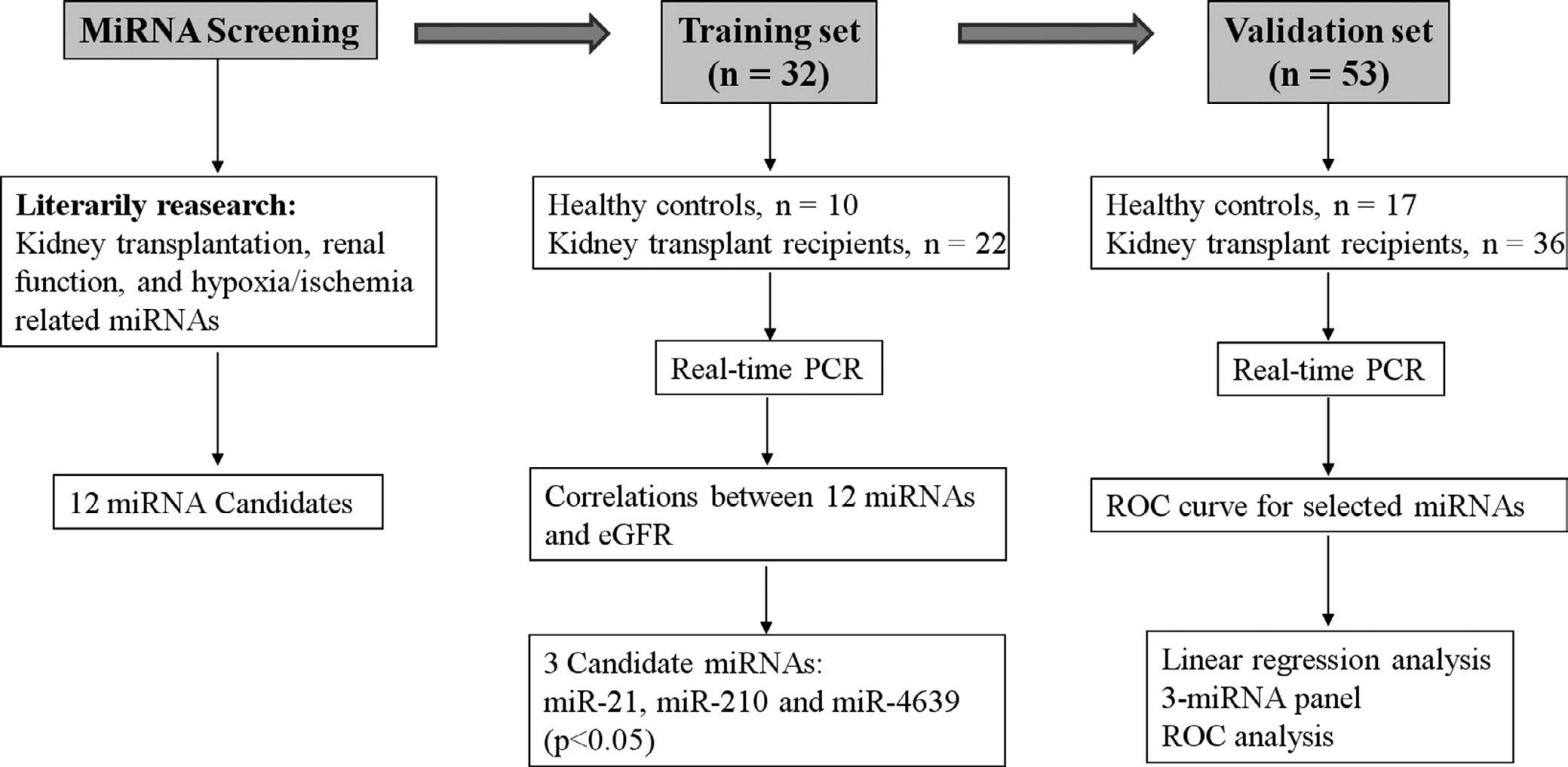
An overview of the experimental design

In this study, we examined correlations between exosomal miRNA levels and eGFR in cohorts of kidney transplant recipients and healthy controls. A circulating exosomal miRNA panel was established as the non‐invasive biomarker for monitoring of post‐transplant renal graft function in the 1‐year follow‐up. The flow chart for the study design is illustrated in Figure 1.

## 2 MATERIALS AND METHODS

### 2.1 Patients and samples

A total of 58 kidney transplant recipients and 27 healthy controls were enrolled in the study (Figure 1). Participants were enrolled between January 2017 and October 2018 in Third Hospital of Soochow University (Changzhou, China). No living donors, HIV‐positive patients and/or re‐transplant patients were included. The allograft function was evaluated by eGFR, which is calculated as the “CKD‐EPI equation”.^19^ For the screening of 12 exosomal miRNAs and the validation of three exosomal miRNAs, patients' plasma samples were collected 3 months after renal transplantation (at study entry). In the follow‐up study, plasma samples were collected at months 3, 6 and 12 after study entry. Two millilitre blood samples were collected from all patients and healthy controls without breakfast in the early morning. Within 2 hours, plasma separation was accomplished by centrifugation at 3200 *g* for 5 minutes to completely remove cell debris. The supernatant plasma was collected and stored at −80°C until analysis. The study was approved by the ethics committee of Soochow University. Written informed consent was obtained from all patients. Basic characteristics of participants are shown in Table 1.

**TABLE 1:** Basic characteristics of participants

	Kidney transplant recipients (n = 58)	Healthy controls (n = 27)
Age (years)	40.17 (9.73)	36.00 (7.39)
Gender		
Male, n (%)	37 (63.79)	13 (48.15)
Female, n (%)	21 (36.21)	14 (51.85)
Blood glucose (mmol/L)	5.42 (0.84)	5.22 (0.90)
Serum creatinine (μmol/L)	140.40 (94.39)	72.59 (13.69)
eGFR (mL/min/1.73 m^2^)	61.84 (20.90)	109.6 (18.83)
Carbamide (mmol/L)	8.58 (4.15)	4.98 (1.15)
Uric acid (μmol/L)	357.70 (97.44)	313.5 (86.98)
Haemoglobin (g/L)	134.00 (19.72)	144.80 (18.58)

Note

Data were presented as the mean (SD).

Abbreviation: eGFR, estimated glomerular filtration rate.

### 2.2 Exosome extraction

Plasma exosomes were extracted by gel exclusion chromatography (Exo‐spin™; Cell Guidance Systems), strictly in accordance with kit instructions. Briefly, 200 μL plasma was centrifuged at 20 000 *g* for 30 minutes to remove cell debris. Supernatant was transferred to a new centrifuge tube and ½ volume of Exo‐spin™ buffer was added. After incubating at 4°C for at least 1 hour, the mixture was centrifuged at 20 000 *g* for 1 hour. Plasma exosomes were re‐suspended in 100 μL phosphate‐buffered saline (PBS), transferred to the top of the Exo‐spin column and centrifuged at 50 *g* for 60 seconds. Eluate was discarded, and additional 200 µL PBS was added to the top of the column. The purified plasma exosomes were harvested in the eluate by centrifuging at 50 *g* for 60 seconds.

### 2.3 Characterization of plasma exosomes

Plasma exosomes were applied to 200‐mesh nickel grids and precipitated for several minutes. Samples were stained with 2% phosphotungstic acid for 1 minute. After drying at room temperature for several minutes, exosomes were imaged by a transmission electron microscope (H‐7650; Hitachi High‐Tech) at 80 kV. The particle size analysis of exosomes was detected by Nanoparticle Tracking System (ZetaView Particle Metrix). Exosomal protein was extracted by RIPA buffer with protease inhibitor (Solarbio). Lysates were boiled in 4 × SDS loading buffer, and the samples were separated by SDS‐PAGE, transferred to a PVDF membrane and detected by immunoblotting analysis with the indicated antibodies using Immobilon Western Chemiluminescent HRP Substrate (Millipore Corp). The following primary antibodies were used: mouse anti‐Alix (1:1000; Cell Signaling Technology), rabbit anti‐CD63 (1:1000; Proteintech), rabbit anti‐CD81 (1:1000; Abcam) and rabbit anti‐calnexin (1:1000; Cell Signaling Technology).

### 2.4 RNA isolation and quantification

Exosomal miRNAs were extracted using TRIzol LS reagent (Invitrogen) according to the instructions of the manufacturer. For miRNA quantification, Bulge‐loop miRNA qRT‐PCR Primer Sets (one miRNA‐specific RT primer and a pair of qPCR primers for each set) specific for each miRNA were designed by RiboBio (Patent No. CN 103740842A). The cDNA was synthesized using PrimeScript™ RT reagent Kit with gDNA Eraser (Takara Biomedical Technology). The qRT‐PCR assay was conducted by TB Green™ Premix Ex Taq™ (Takara Biomedical Technology). qRT‐PCR was conducted on ABI 7500 system (Applied Biosystems). The miR‐16 expression was used as endogenous control because it is consistently expressed in exosomes from plasma samples. Relative miRNA expression was calculated by the $2^{‐\Delta C_{t}}$ method in which ΔC*_t_* was calculated as C*_t_* (miRNA of interest) − C*_t_* (reference gene).

### 2.5 Statistical analysis

Data analysis was conducted by SPSS 19.0 statistical software (IBM Corp.) and GraphPad Prism 7 software (GraphPad Software, Inc). Data are expressed as the mean ± SD, number (percentage) or median (10%‐90% percentiles) when appropriate. Correlations between variables were calculated using Spearman's rank‐order correlations, and the diagnostic performance of biomarkers was evaluated by ROC curves. All *P*‐values were two‐tailed and *P* < .05 was considered to indicate a statistically significant difference.

## 3 RESULTS

### 3.1 Identification of plasma exosomes

**FIGURE 2:**
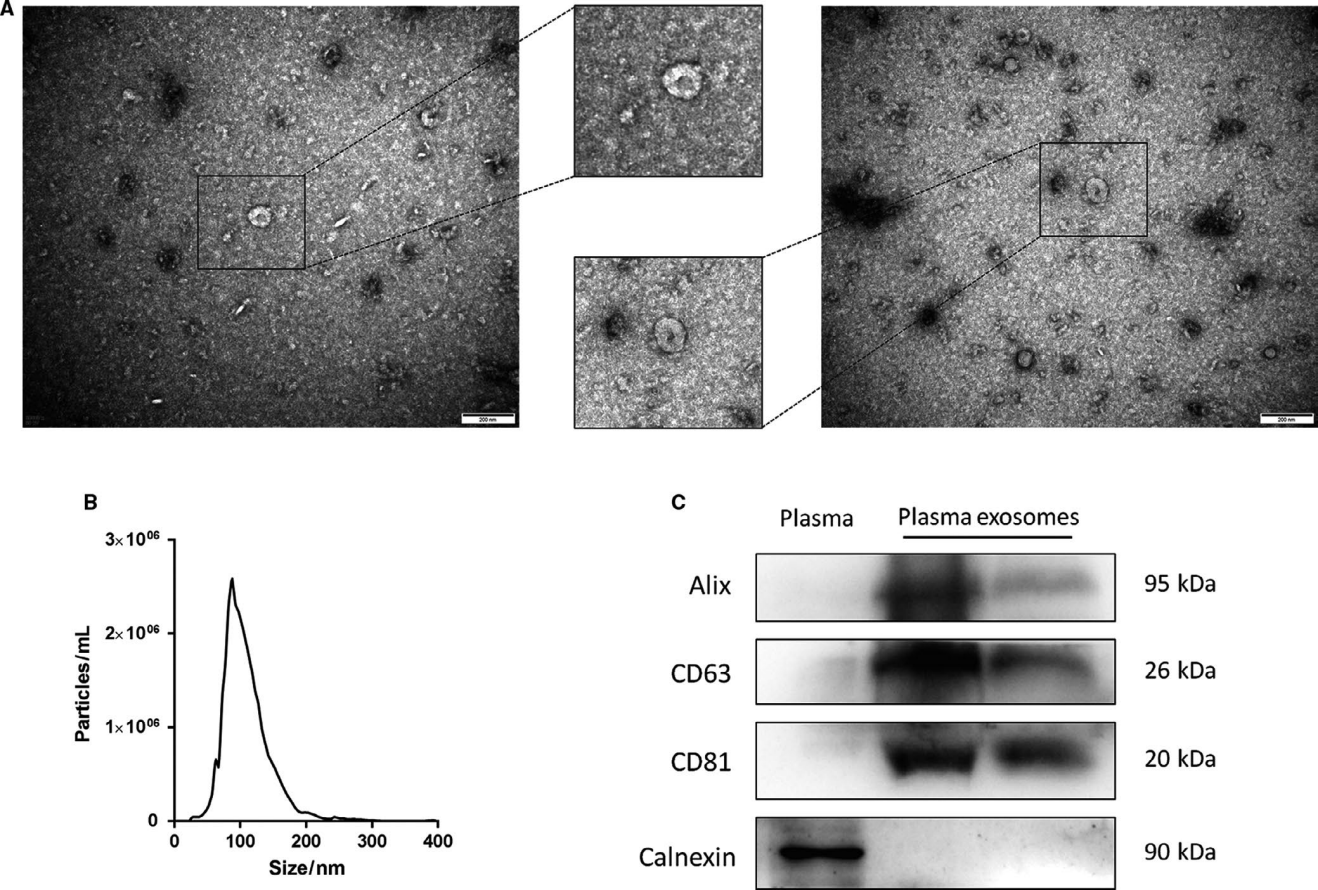
Characterization of plasma exosomes. A, Representative electron micrograph of plasma exosomes. B, Size distribution of plasma exosomes analysed by nanoparticle tracking system. C, Western blot analysis of common exosomal markers Alix, CD63 and CD81, and the endoplasmic reticulum marker calnexin. Plasma was used as a control.

Plasma exosomes were characterized by transmission electron microscopy, nanoparticle tracking analysis and Western blotting. Transmission electron microscopy showed typical size and morphology of exosomes (Figure 2A). Nanoparticle tracking analysis confirmed the homogeneous size of vesicles with ~ 100 nm diameters (Figure 2B). The average concentration was (9.08 ± 0.45) × 10^10^ particles/mL. The isolated exosomes had detectable Alix, CD63 and CD81, three established markers for exosomes. They had no expression of the endoplasmic reticulum marker calnexin, which served as a negative exosomal marker (Figure 2C).

### 3.2 Correlations between exosomal miRNA levels and eGFR

As illustrated in Figure 1, a literature search for miRNAs which related to kidney transplantation, renal function and hypoxia/ischaemia conditions was conducted. Combined with our previous miRNA‐sequencing data^20^ and the endogenous miRNA expression levels, 12 miRNAs were selected as candidates for validation in individual plasma samples in a training set. The training set included 22 kidney transplant recipients and 10 healthy controls. RT‐qPCR assay was used to measure relative miRNA levels in plasma exosomes, and eGFR levels were calculated for each individual. Exosomal expression levels of the 12 miRNAs in kidney transplant recipients compared to healthy controls were presented in Figure S1. Pearson's correlation coefficients between exosomal miRNAs and eGFR were summarized in Table 2.

**TABLE 2:** Pearson's correlation coefficients for the associations between exosomal miRNAs and eGFR in the training set

Exosomal miRNAs	Pearson's *r*	*P*‐value
let‐7c‐5p	−.1440	.4166
miR‐20a‐5p	.1365	.2634
miR‐21‐5p	−.4178	**.0173**
miR‐24‐3p	−.0943	.4376
miR‐29b‐3p	.0785	.6802
miR‐30c‐5p	−.3300	.0566
miR‐34a‐5p	−.3050	.0794
miR‐146a‐5p	−.2596	.1382
miR‐192‐5p	−.0219	.9086
miR‐199a‐5p	.0886	.6415
miR‐210‐3p	−.3860	**.0139**
miR‐4639‐5p	−.4052	**.0214**

Note

*P*‐value <.05 was defined as statistically significant and showed in bold values.

The training set (n = 32).

Abbreviation: eGFR, estimated glomerular filtration rate.

**FIGURE 3:**
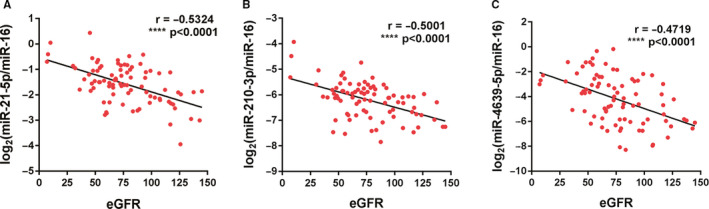
Correlations of eGFR and miRNA expression in plasma exosomes. A, miR‐21‐5p; B, miR‐210‐3p; C, miR‐4639‐5p. Pearson's correlation coefficient (rho) is shown. eGFR, estimated glomerular filtration rate. ****P < .0001

Based on the analyses of the training set, three different exosomal miRNAs (miR‐21‐5p, miR‐210‐3p and miR‐4639‐5p) correlated significantly with eGFR (Table 2). Thus, these three miRNAs were further examined by qRT‐PCR in a larger cohort of validation set including 36 kidney transplant recipients and 17 matched healthy controls. Consistent with the results from the training set, miR‐21‐5p, miR‐210‐3p and miR‐4639‐5p were found to be correlated with eGFR. Figure 3 showed negative correlations between the log‐transformed expression of three exosomal miRNAs and eGFR in the entire sets (all individuals in the training and validation sets: 58 kidney transplant recipients and 27 healthy controls; *r* = −.5324, −.5001, −.4719, respectively, and *P* < .0001). It indicated that expression levels of exosomal miRNA levels in plasma were associated with eGFR and renal function.

### 3.3 Diagnostic potential of individual exosomal miRNA

**FIGURE 4:**
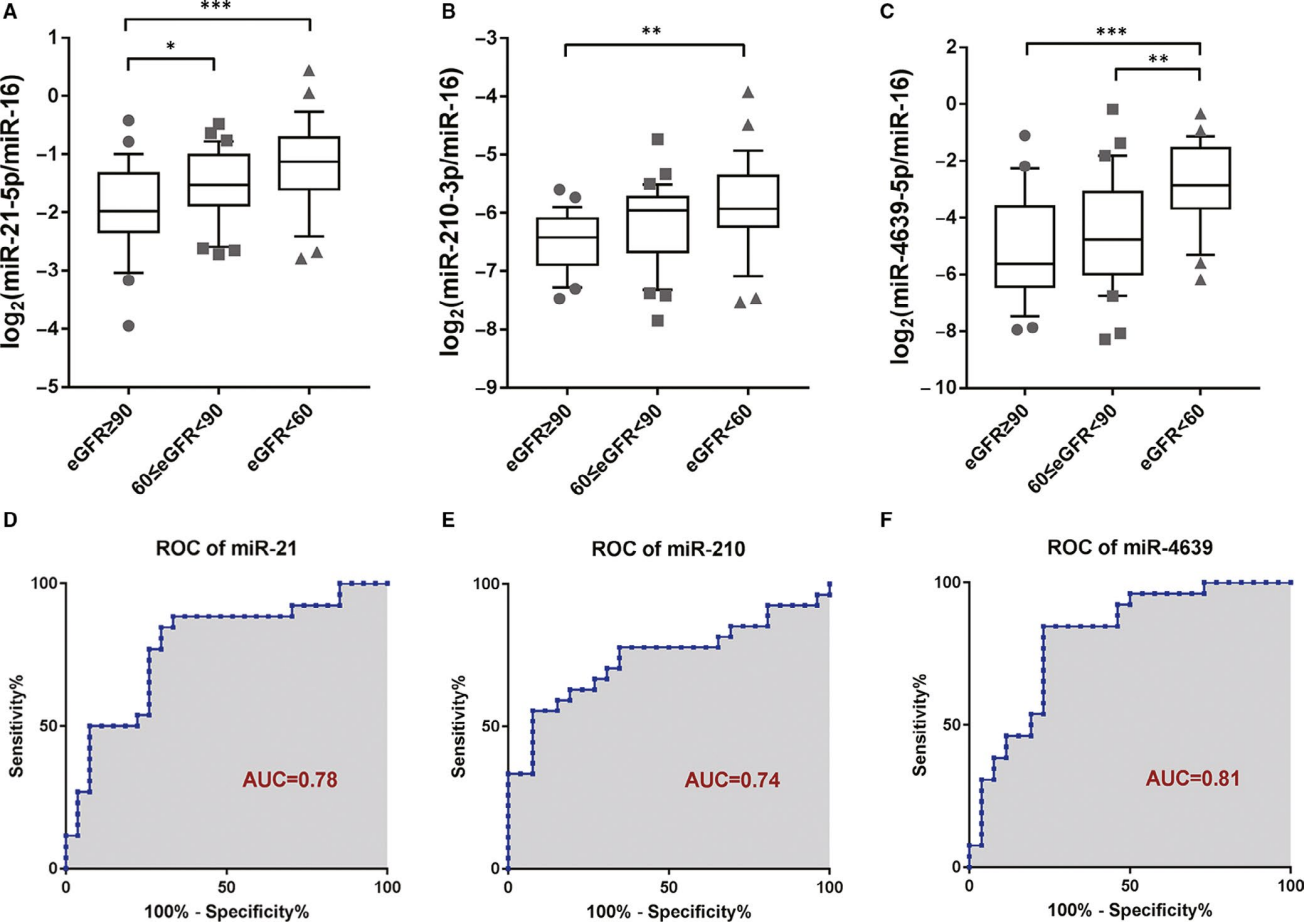
Discrimination of eGFR level by exosomal miRNA expression. A‐C, Relative expression levels of miR‐21‐5p, miR‐210‐3p and miR‐4639‐5p in participants with different eGFR levels. Boxes represented the interquartile range of the data. The lines across the boxes and the numbers indicated the median values. The hash marks above and below the boxes indicated the 90th and 10th percentiles for each group, respectively. eGFR ≥ 90 (mL/min/1.73 m^2^): n = 26; 60 ≤ eGFR <90 (mL/min/1.73 m^2^): n = 33; eGFR < 60 (mL/min/1.73 m^2^): n = 26. D‐F, ROC curve analysis of exosomal miR‐21‐5p, miR‐210‐3p and miR‐4639‐5p in distinguishing subjects with eGFR < 60 (mL/min/1.73 m^2^) from those with eGFR ≥ 90 (mL/min/1.73 m^2^). eGFR, estimated glomerular filtration rate; ROC curve, receiver operating characteristic curve; AUC, area under the curve. *P < .05, **P < .01, ***P < .001

Participants of the entire sets (n = 85) were divided into three groups according to their eGFR levels: eGFR < 60, 60 ≤ eGFR <90, and eGFR ≥ 90 (mL/min/1.73 m^2^). The number of individuals in each group was 26, 33 and 26, respectively. Relative expression levels of miR‐21‐5p, miR‐210‐3p and miR‐4639‐5p in plasma exosomes were significantly higher in transplant recipients with chronic allograft dysfunction (eGFR < 60 mL/min/1.73 m^2^) than in those with normal graft function (eGFR > 90 mL/min/1.73 m^2^) (Figure 4A‐C). Moreover, exosomal miR‐21‐5p exhibited a significantly different expression between groups of eGFR ≥ 90 and 60 ≤ eGFR <90 (*P* < .05, Figure 4A). Exosomal miR‐4639‐5p exhibited a significantly different expression between groups of 60 ≤ eGFR <90 and eGFR < 60 (*P* < .01, Figure 4C).

To evaluate whether these three selected exosomal miRNAs had the potential to evaluate renal function, ROC curves were constructed with the individuals of chronic allograft dysfunction (eGFR < 60 mL/min/1.73 m^2^) and normal graft function (eGFR > 90 mL/min/1.73 m^2^). The areas under the ROC curves (AUC) of miR‐21‐5p, miR‐210‐3p and miR‐4639‐5p were 0.78, 0.74 and 0.81, respectively (Figure 4D‐F). Using the optimal cut‐off values obtained from ROC curves of exosomal miRNAs, sensitivities ranging from 55.56% to 88.46% and specificities of 66.67% to 92.31% were obtained (Table 3). These results suggested that exosomal miR‐21‐5p, miR‐210‐3p and miR‐4639‐5p may have potential for monitoring renal function.

**TABLE 3:** Summarized diagnostic factors of the individual miRNA and combined miRNA panels

	Value (mean, SD)		Fold change (eGFR ≥ 90/eGFR < 60)	Cut‐off value	AUC (95% CI)	Sensitivity (%)	Specificity (%)
	eGFR ≥ 90	eGFR < 60					
log_2_(miR‐21/miR‐16)	−1.98 (0.78)	−1.21 (0.75)	1.64	>−1.85	0.78 (0.65‐0.91)	88.46	66.67
log_2_(miR‐210/miR‐16)	−6.52 (0.52)	−5.93 (0.83)	1.10	>−5.96	0.74 (0.60‐0.88)	55.56	92.31
log_2_(miR‐4639/miR‐16)	−5.03 (1.87)	−2.89 (1.48)	1.74	>−3.77	0.81 (0.69‐0.93)	84.62	76.92
miR‐21 + 210 (score)	0.35 (0.22)	0.65 (0.24)	0.54	>0.46	0.83 (0.71‐0.95)	88.46	73.08
miR‐21 + 4639 (score)	0.28 (0.27)	0.72 (0.25)	0.39	>0.42	0.88 (0.79‐0.97)	88.46	73.08
miR‐210 + 4639 (score)	0.33 (0.28)	0.67 (0.21)	0.49	>0.51	0.82 (0.71‐0.93)	80.77	73.08
miR‐21 + 210 + 4639 (score)	0.28 (0.27)	0.72 (0.25)	0.39	>0.43	0.89 (0.80‐0.97)	88.46	73.08

Abbreviations: AUC, area under the curve; CI, confidence interval; eGFR, estimated glomerular filtration rate (mL/min/1.73 m^2^); ROC curve, receiver operating characteristic curve.

### 3.4 Establishment of a predictive diagnostic miRNA panel and longitudinal eGFR analysis

MiR‐21‐5p, miR‐210‐3p and miR‐4639‐5p were combined into panels to further evaluate their diagnostic potential for renal function. Logistic regression model was applied to combine exosomal miRNAs into two‐miRNA panels or three‐miRNA panel with the samples from eGFR < 60 and eGFR ≥ 90 (mL/min/1.73 m^2^) groups. The optimal cut‐off values, AUC, 95% confidence intervals (CI), sensitivities and specificities for each analysis were summarized in Table 3.

**FIGURE 5:**
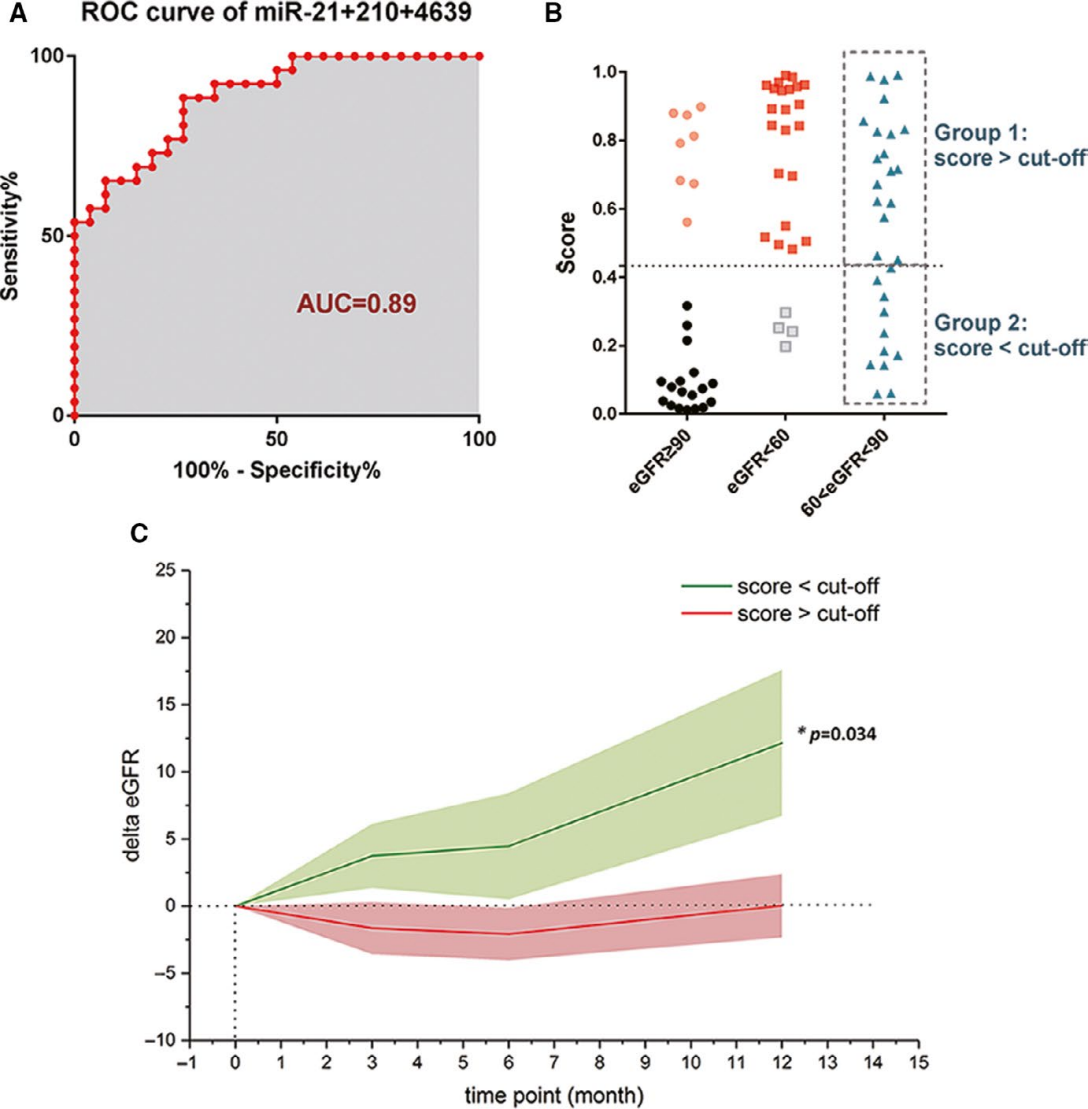
Establishment of a predictive diagnostic miRNA panel and longitudinal eGFR analysis. A, ROC curve of the 3‐miRNA panel in discriminating subjects with eGFR < 60 mL/min/1.73 m^2^ (n = 26) from those with eGFR ≥ 90 mL/min/1.73 m^2^ (n = 26) in the training and validation sets. B, Dot plot presenting the distributions of scores generated from 3‐miRNA panel in discriminating kidney transplant recipients with different eGFR levels. Scores ranging from 0 to 1 were generated for each sample according to logistic regression equation. C, Association between scores generated from 3‐miRNA panel and Δ eGFR in longitudinal analysis. Kidney transplant recipients with 60 ≤ eGFR <90 (mL/min/1.73 m^2^) were divided into two groups according to the optimal cut‐off value of 3‐miRNA panel. The change of eGFR levels in the following 12 mo was presented (mean ± SEM). eGFR, estimated glomerular filtration rate; ROC curve, receiver operating characteristic curve; AUC, area under the curve; *P < .05

ROC analysis demonstrated that the three‐miRNA panel exhibited increased sensitivity and specificity in discriminating between transplant recipients with chronic allograft dysfunction (eGFR < 60 mL/min/1.73 m^2^) and those with normal graft function (eGFR > 90 mL/min/1.73 m^2^), as compared with individual miRNA or two‐miRNA panels (Table 3). Algorithms of three‐miRNA panel were built by logistic regression and were calculated from the following equation:

Logit(P)=6.644+1.533×[miR‐21‐5p]+0.200×[miR‐210‐3p]+0.752×[miR‐4639‐5p]

(log‐transformed expression of exosomal miRNAs was used in square brackets). When using the optimal cut‐off value of 0.43, the diagnostic sensitivity and specificity of the three‐miRNA panel were 88.46% and 73.08%, respectively, and the AUC was 0.89 (95% CI, 0.80‐0.97) (Figure 5A,B).

We further evaluated the usefulness of the above three‐miRNA panel for monitoring post‐transplant renal graft function in longitudinal analysis. Kidney transplant recipients with 60 ≤ eGFR <90 (mL/min/1.73 m^2^) were further divided into two groups according to the calculated score of three‐miRNA panel. The group 1 was defined as those with a score above the optimal cut‐off value (>0.43, n = 18), and the group 2 was defined as those with a score below the optimal cut‐off value (<0.43, n = 11) (Figure 5B). Then, the changing rates of eGFR levels were compared between the two groups in the following 12 months. The eGFR level of each individual was collected at the time‐point of 3, 6 and 12 months during follow‐up. We found that individuals in group 2 (calculated score <cut‐off) had significantly elevated eGFR levels compared with those in group 1 (calculated score >cut‐off) (Figure 5C). Our longitudinal analysis implied that the score of three‐miRNA panel may predict future eGFR recovery and the improvement of post‐transplant renal graft function.

## 4 DISCUSSION

In the past 20 years, with the application of highly effective immunosuppressive drugs, major progress has been made in extending graft and patients' survival after kidney transplantation. Nevertheless, long‐term graft survival is still suboptimal due to both immunologic and non‐immunologic factors, including ischaemia/reperfusion injury, untreated or ineffective clinical and subclinical rejection, nephrotoxicity of calcineurin inhibitors and existed donor diseases.^3^, ^4^ Therefore, it is imperative to investigate specific and non‐invasive biomarkers for continuous monitoring post‐transplant renal graft function, which may help to predict disease progression and determine therapeutic strategies.

Exosomes are tiny vesicles released from cells and widely found in body fluids such as blood, urine and saliva.^16^ Accumulating evidence has demonstrated that exosomes contain a large number of molecules including protein, lipids, mRNAs and miRNAs.^15^, ^21^ These molecules carry a large amount of intracellular biological information that is closely related to disease status.^14^, ^22^ Due to complete membrane structures, exosomes are less disturbed by the external environment and carry small molecules with good stability.^23^ Thus, molecules in exosomes such as miRNAs can be referred as non‐invasive biomarkers for the detection of renal diseases.^16^ Indeed, exosomal miRNAs have been suggested to participate in pathogenesis of different renal diseases and serve as disease biomarkers, including tubulointerstitial inflammation,^24^, ^25^ renal fibrosis,^26^, ^27^, ^28^ ischaemic kidney injury,^29^, ^30^ IgA nephropathy,^31^ acute kidney injury ^32^, ^33^ and chronic kidney disease.^34^ However, the use of circulating exosomal miRNAs for monitoring post‐transplant renal graft function has not yet been further explored.

In the present study, we examined 12 different exosomal miRNAs according to the literature search and our previous miRNA‐sequencing data.^20^ All miRNAs are related to kidney transplantation, renal function and hypoxia/ischaemia conditions. Three different miRNAs (miR‐21, miR‐210 and miR‐4639) showed significant negative correlations with eGFR. MiR‐21 is a hypoxia/ischaemia‐sensitive miRNA that play important role in modulating renal function. Khalid et al reported a predictive value of miR‐21 combined with other five miRNAs for delayed graft function following kidney transplantation.^35^ Urinary exosomal miR‐21 was reported to be significantly up‐regulated in patients with diabetic kidney disease,^36^ chronic kidney disease and after glomerular injury.^37^ These results were consistent with our observations that patients with impaired renal function tended to have elevated miR‐21 expression in plasma exosomes.

MiR‐210 is another hypoxia/ischaemia‐associated miRNA that regulates cellular events in the kidney by targeting multiple genes. Lorenzen et al showed that miR‐210 level was strongly altered in urine of the patients with acute renal allograft rejection.^38^ Deregulated miR‐210 level was associated with higher decline in GFR after 1‐year transplantation.^38^ It is also reported that circulating miR‐210 could predict survival in critically ill patients with acute kidney injury,^39^ indicating a clinical application of miR‐210 in disease monitoring. MiR‐4639 is a newly discovered miRNA that participates in cellular oxidative stress responses.^40^ MiR‐4639 is enriched in exosomes of human plasma that may facilitate biomarker discovery.

In the present study, the combination of three miRNAs as a panel exhibited a better diagnostic potential compared with individual miRNA or two‐miRNA panels (Table 3). The purpose of establishing the miRNA panel and 1‐year follow‐up was to examine the potential value of this panel for monitoring and predicting post‐transplant renal graft function, and further favour disease treatment. Kidney transplant recipients with 60 ≤ eGFR <90 mL/min/1.73 m^2^ have only slight or mild renal injury that cannot be diagnosed as chronic allograft dysfunction clinically, thus were enrolled in the longitudinal study. By evaluating expression level of exosomal miRNAs 3 months after renal transplantation, we can predict the disease progression of recipients with 60 ≤ eGFR <90 mL/min/1.73 m^2^ according to the predictive score of this panel. Recipients with high predictive score (high risk) were associated with poor prognosis, even progressive renal function deterioration. Therefore, doctors were able to identify the causes and pathological changes of these patients in time. Current immunosuppressive protocols can also be adjusted properly to improve long‐term renal allograft outcome for these patients. By contrast, recipients with low predictive score (low risk) were associated with long‐term elevation of eGFR, indicating the stable recovery of allograft function. They can maintain the original immunosuppressive protocols and may need no additional medications. Therefore, the exosomal miRNA panel had the ability to predict disease progression, and instructive for disease prevention and treatment.

The ideal biomarker for kidney transplant recipients should provide sensitive and accurate monitoring of graft function, early and specific diagnosis of rejection and the assessment of long‐term outcome in a non‐invasive, cost‐effective manner.^41^ Most of the exosomes in plasma originate from the cell types in contact with the vascular lumen, including blood cells and endothelial cells.^42^ Similarly, urinary exosomes are mostly derived from cells in contact with the renal tubule lumen, such as renal tubular epithelial cells.^43^ Thus, urine is also an appropriate non‐invasive biofluid for exosomal studies. However, by deep sequencing analysis, Lesley Cheng et al found that the number and abundance of miRNAs in cell‐free urine exosomes were significantly lower than those in plasma exosomes.^44^ Only 12 miRNAs were abundantly expressed from 2.5 mL of cell‐free urine, while 1 mL of plasma can contain more than 500 high‐abundance miRNAs.^45^, ^46^ This phenomenon may be related to high RNase activity in the bladder.^47^ Moreover, miRNAs in plasma exosomes can be accurate quantified by controlling sample volume and detecting miRNA internal control gene. However, the amount of exosomes in urine can be easily affected by the water intake of the body. Due to the difference in the composition of morning urine samples and urine samples at other time‐points, morning urine samples should be obtained for analysis, which may bring inconvenience. Therefore, in this study, plasma was used as the source for isolating exosomal miRNAs for further analysis.

The exosomal miRNA panel proposed in this study has some advantages comparing to the existing methods. First, examine exosomes isolated from plasma is a non‐invasive procedure. Second, due to complete membrane structures of exosomes, miRNAs packed in exosomes are relatively stable and not easily influenced by external factors. Third, the three‐miRNA panel is sensitive in discriminating between transplant recipients with chronic allograft dysfunction and those with normal graft function. Moreover, exosomal miRNA panel has the ability to predict long‐term graft function in our longitudinal analysis. However, this exosomal panel also has some limitations. Isolating exosomes from plasma samples and qRT‐PCR analysis are relatively expensive compared to Cr or eGFR testing. Exosome extraction assays are not available in most clinical laboratories. In addition, the present study included only relatively small populations and only 1‐year follow‐up. Thus, future investigations of larger sample size from multi‐centres are needed before the circulating exosomal miRNA panel can be used in clinical applications. Also, it is our interest to explore the underlying mechanism of deregulated exosomal miRNAs and post‐transplant renal graft function.

## 5 CONCLUSIONS

In summary, this work revealed that miR‐21, miR‐210 and miR‐4639 in plasma exosomes correlate closely with eGFR. The diagnostic value of the joint exosomal miRNA panel based on miR‐21, miR‐210 and miR‐4639 was superior to single or double indicators. Longitudinal eGFR analysis further demonstrated the usefulness of exosomal miRNA panel as a non‐invasive biomarker for monitoring post‐transplant renal graft function.

## CONFLICT OF INTEREST

The authors confirm that there are no conflicts of interest. The authors had full access to all of the data in this study and take complete responsibility for the integrity of the data and the accuracy of the data analysis.

## AUTHOR CONTRIBUTIONS

**Yimeng Chen:** Investigation (lead); Methodology (lead); Project administration (equal); Validation (lead); Writing‐original draft (lead); Writing‐review & editing (lead). **Xu Han:** Investigation (equal); Validation (equal); Writing‐original draft (equal); Writing‐review & editing (equal). **Yangyang Sun:** Resources (lead); Supervision (equal); Validation (equal); Writing‐review & editing (supporting). **Xiaozhou He:** Funding acquisition (equal); Project administration (equal); Supervision (equal); Writing‐review & editing (equal). **Dong Xue:** Funding acquisition (lead); Project administration (lead); Resources (equal); Validation (equal); Writing‐original draft (equal); Writing‐review & editing (equal).

## ETHICAL APPROVAL

The study was approved by the ethics committee of Soochow University. Informed consents were obtained from all participants.

## Supporting information

Fig S1

## Data Availability

The data that support the findings of this study are available from the corresponding author upon reasonable request.
