# Supplementary material for: A circulating exosomal microRNA panel as a novel biomarker for monitoring post‐transplant renal graft function
Source: J Cell Mol Med. 2020 Sep 11;24(20):12154–63. doi: 10.1111/jcmm.15861 (PMC7579686; doi:10.1111/jcmm.15861)
Supplement: Supplementary file 1 — Fig S1 [file JCMM-24-12154-s001.docx]

**Supplementary Figure 1**

**Exosomal miRNA expression levels of healthy controls and kidney transplant recipients.**


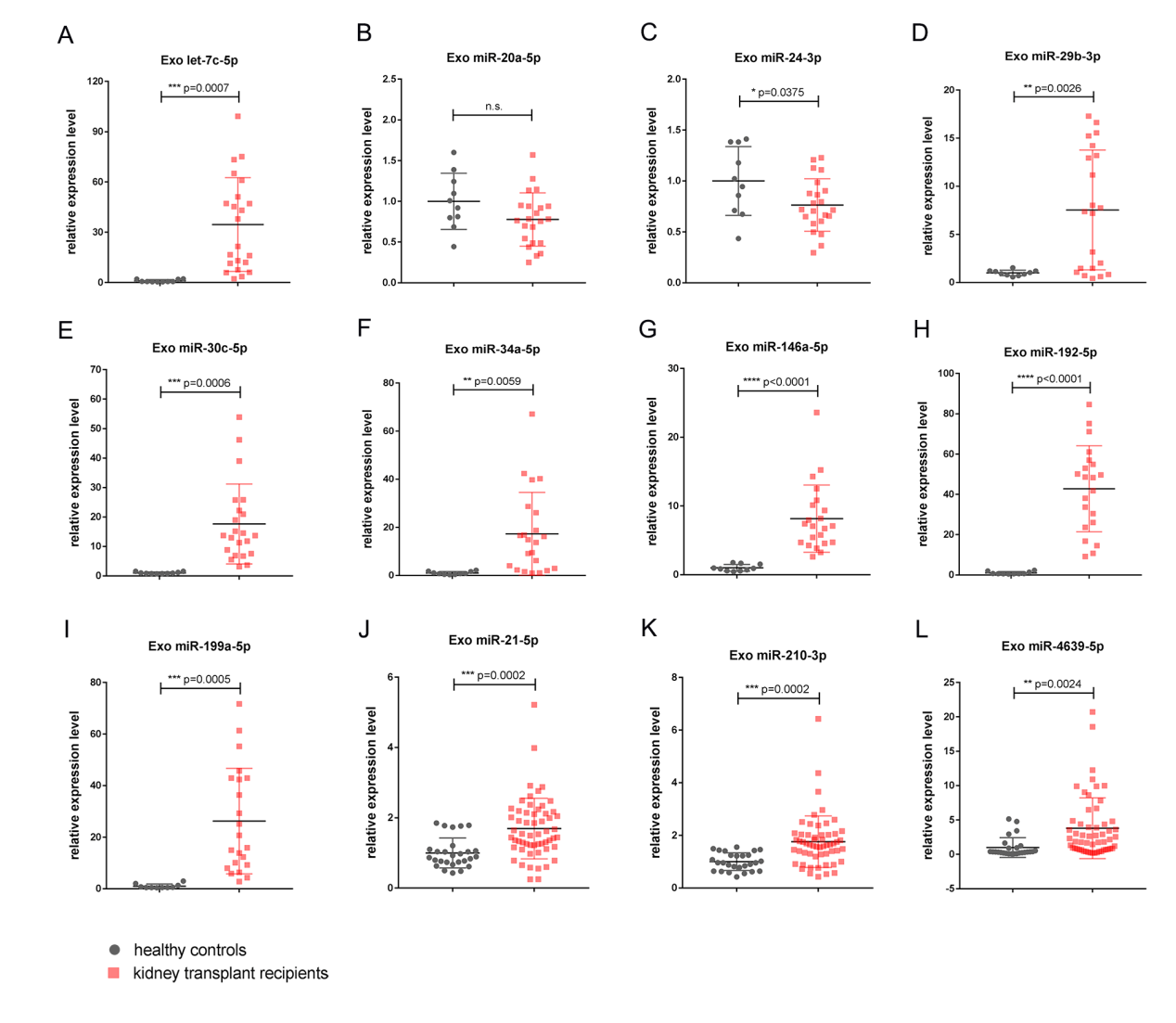


(A-I) Dot plots represent relative expression levels of 9 plasma exosomal miRNAs in the training set including 10 healthy controls and 22 kidney transplant recipients.

(J-L) Dot plots represent relative expression levels of plasma exosomal miR-21-5p, miR-210-3p, and miR-4639-5p in the training and validation set including 27 healthy controls and 58 kidney transplant recipients. * p < 0.05, ** p < 0.01, *** p < 0.001.
